# Genetic Variation in *POU4F3* and *GRHL2* Associated with Noise-Induced Hearing Loss in Chinese Population: A Case-Control Study

**DOI:** 10.3390/ijerph13060561

**Published:** 2016-06-03

**Authors:** Xiangrong Xu, Qiuyue Yang, Jie Jiao, Lihua He, Shanfa Yu, Jingjing Wang, Guizhen Gu, Guoshun Chen, Wenhui Zhou, Hui Wu, Yanhong Li, Huanling Zhang

**Affiliations:** 1 Department of Occupational and Environmental Health, School of Public Health, Peking University, Beijing 100191, China; selina6887@163.com (X.X.); yqy9009@126.com (Q.Y.); 1511210232@bjmu.edu.cn (J.W.); 2 Henan Provincial Institute for Occupational Health, Zhengzhou 450052, Henan, China; jiaojie66@126.com (J.J.); guizhengu@163.com (G.G.); zhouwh2008@163.com (W.Z.); wuhui2009yan@163.com (H.W.); liyanhong_sky@126.com (Y.L.); 3 Wugang Institute for Occupational Health, Wugang 462500, Henan, China; guoshunchen@126.com (G.C.); zhanghuanlingw@163.com (H.Z.)

**Keywords:** NIHL, *POU4F3*, *GRHL2*, noise exposure, genetic susceptibility

## Abstract

Noise-induced hearing loss (NIHL) is an important occupational disease worldwide resulting from interactions between genetic and environmental factors. The purpose of this study was to examine whether genetic variations in *POU4F3* and *GRHL2* may influence susceptibility to NIHL in the Chinese population. A matched case-control study was carried out among 293 hearing loss individuals and 293 normal hearing workers drawn from a population of 3790 noise-exposed workers. Ten single-nucleotide polymorphisms (SNPs) in *POU4F3* and *GRHL2* were selected and genotyped. Logistic regression was performed to analyze the main effects of SNPs and the interactions between noise exposure and SNPs. Moreover, the interactions between predictor haplotypes and noise exposure were also analyzed. Analysis revealed that the CC genotype of rs1981361 in the *GRHL2* gene was associated with a higher risk of NIHL (adjusted OR = 1.59; 95% CI: 1.08–2.32, *p* = 0.018). Additionally, the GG genotype of rs3735715 in the *GRHL2* gene was also a risk genotype (adjusted OR = 1.48; 95% CI: 1.01–2.19, *p* = 0.046). Significant interactions were found between rs3735715, rs1981361 (*GRHL2*), rs1368402 as well as rs891969 (*POU4F3*) and noise exposure in the high-level exposure groups. Furthermore, the protective haplotype CA in the *POU4F3* gene and the risk haplotype GCCG in the *GRHL2* gene were identified combined with noise exposure. These results indicated that *GRHL2* might be an NIHL susceptibility gene, but the effect of *POU4F3* on NIHL could only be detected when taking noise exposure into account, and their effects were enhanced by higher levels of noise exposure. However, the differences were not significant after the Bonferroni correction was applied. These results should be seen as suggestive.

## 1. Introduction

Noise-induced hearing loss (NIHL) is a sensorineural hearing deficit which can occur from exposure to intermittent or continuous loud noise, and it can damage the sensory hair cells located in the cochlea, which results in permanent hearing loss [1]. NIHL is a worldwide leading occupational health risk, especially in industrialized countries, and it is the second most common form of sensorineural hearing impairment [2]. NIHL is a complex disorder, induced by a combination of environmental and genetic factors. The responsible environmental factors have been well studied, including noise, organic solvents, heat, vibrations, smoking and drinking [3,4,5]. However, the degree of hearing loss varies widely among workers exposing to similar levels of noise and other environmental factors [6]. Additionally, animal studies exhibited that genetic factors could influence individual susceptibility to noise. From the candidate gene association studies, some predisposing genes have been confirmed that might modify the susceptibility to NIHL, such as oxidative stress genes (*GSTM1*, *PON2*, *SOD2*, *CAT*), potassium ions recycling genes (*KCNE1*, *KCNQ4*, *KCNQ1*, *GJB1*, *GJB2*, *GJB4*, *KCNJ10*), monogenic deafness genes (*PCDH15*, *MYH14*), and heat shock protein genes (*HSP70*) [2]. These results indicated that genetic variations might play important roles in the development of NIHL.

The POU-domain transcription POU4F3 is a member of the POU-domain class IV transcription factor family. The POU4F3 protein plays an important role in the maturation, differentiation and survival of hair cells [7]. In the inner ear, *POU4F3* is uniquely and strongly expressed in cochlear and vestibular hair cells. Targeted deletion of *POU4F3* results in profound deafness and impaired balance due to complete loss of auditory and vestibular hair cells [8]. A mutation in the *POU4F3* gene is associated with autosomal dominant late-onset progressive hearing loss (DFNA15) in several families [9,10]. To our best knowledge, only one study by Konings *et al.* [11] described the positive interaction between *POU4F3* rs891969 and noise exposure on the risk of NIHL in the Swedish population. To date, no evidence indicates that *POU4F3* is the gene susceptible to NIHL in the Asian population.

Grainyhead-like2 (GRHL2) is a member of the grainyhead-like (GRHL) transcription factor family, which is widely expressed in cochlear duct lining cells. This family of transcription factors controls the development and differentiation of multicellular epithelia by regulating genes germane to cell junction formation and proliferation [12,13]. The ion channels and junction proteins in the otic epithelial cells play an improtant role in homeostasis maintenance and inner-ear development. In the animal experiments, *GRHL2* knockout was embryonically lethal in mice, displaying neural tube and split face [14]. *GRHL2* mutant zebrafish showed inner-ear defects and abnormal swimming positions. The inner-ear defects in the zebrafish were rescued by injecting wild-type human *GRHL2* mRNA [15]. The human *GRHL2* mutation leads to non-syndromic sensorineural deafness autosomal dominant type 28 (DFNA28) [7,9]. Other polymorphic sequence variants in *GRHL2* have been implicated in age-related hearing impairment [16]. So far, only a few studies have reported that the *GRHL2* gene might be responsible for the development of NIHL [17,18].

Considering the important roles of the *POU4F3* and *GRHL2* genes in the auditory system, we assumed that genetic variability in these two genes might be associated with susceptibility to NIHL. To examine this hypothesis, we investigated the 10 SNPs in *POU4F3* and *GRHL2* genes that might account for NIHL development in the Chinese population. Furthermore, we explored the interaction effects among these SNPs and the risk factors.

## 2. Methods

### 2.1. Subjects

This study was performed using the matched case-control method of data collection and analysis. All the subjects were recruited from a cross-sectional survey among 3790 workers who worked in a steel factory of Henan Province in China conducted between September and December in 2013. The hearing level (HL) of all the subjects was examined by pure-tone audiometry (PTA). All the subjects received a physical examination and filled in the questionnaires. Blood samples were also taken for further analysis. The inclusion criteria were that subjects had to be exposed to occupational noise higher than 80 dB and the cumulative time of noise exposure was ≥3 years. The exclusion criteria included: (1) subjects who served as aircraftsman or artillery, had a perforated eardrum, or had a history of explosive noise exposure, skull trauma, a family history of deafness, contagious diseases (mumps, measles and rubella) and treatment with an ototoxic drug (aminoglycoside); (2) subjects with Meniere’s disease, middle ear disease, conductive hearing loss, exaggerated hearing loss, feigned deafness, sudden deafness, toxic deafness, deafness caused by contagious diseases, tumors, autoimmunological diseases and others; (3) subjects whose pure-tone audiogram showed horizontal lines or near-horizontal lines, and subjects who had impairment of HL in linguistic frequency that was more severe than that of HL in high frequency.

Then, subjects were selected and divided into the case group and the control group according to the following criteria. The case group was defined as the average of binaural HL of high frequency ≥40 dB. The individually matched control group was defined as the binaural HL of any frequency <25 dB. The control group was individually matched with a case group by same gender, age (±5 years), type of work and duration of exposure to noise (±2 years) to control environmental confounders. Finally, 239 male cases and 239 male control workers were included as subjects in this study.

All subjects gave their informed consent for inclusion before they participated in the study. The study was approved by the Ethics Committee of Henan Institute of Occupational Medicine (Approval codes: 2013001 and 2013003).

### 2.2. Physical Examination and Questionnaire

A structured questionnaire was administered to the workers along with the physical examination. The questionnaire was designed to collect the following information: age, sex, occupational history (type of work and employment year), lifestyle (smoking and drinking), history of disease, noise exposure at previous workplace, noise exposure in the army, hearing protection measures and so on. Face-to-face interviews were conducted by the trained investigators, and all the physical examinations were performed by the trained physicians. Parameters such as weight, height, pulse rate, systolic and diastolic blood pressure level were also measured following a standard protocol.

### 2.3. Audiological Status Assessment and Environmental Noise Measurement

The audiometry was done using AS216 audiometer (Interacoustics AS Company, Assens, Denmark). All the audiometric tests were performed among 3790 workers with standard procedures in quiet meeting rooms with a background noise level of <25 dB(A) by trained occupational health physician. The data of pure-tone air conduction hearing threshold tests were recorded at the frequencies of 500, 1000, 2000, 3000, 4000, and 6000 Hz after subjects stopped noise exposure for at least 12 h. The averages of 3000, 4000, and 6000 Hz were calculated as the threshold levels at high frequency for each ear. The hearing thresholds at speech frequency were calculated by the average of 500, 1000, and 2000 Hz for each ear. The audiometric raw data were polished by the the confounding effects of age and gender on the basis of the Diagnostic Criteria of Occupational NIHL (Chinese National Criteria GBZ49-2014) [19]. In addition, their ears were inspected according to this standard. Otology morphological examination and otoscopy were requested, including bilateral auricle malformation, external auditory canal malformation and stenosis, tympanic membrane perforation, adhesion or calcification, *etc.*

Based on the Occupational Health Standard of the People’s Republic of China: Measurement of Noise in the Workplace (GBZ/T 189.8-2007) [20], noise exposure levels were assessed from 8 a.m. to 4 p.m. during the subjects’ working time at the representational sites of each type of work using Noise Dose Meters (NoisePro series, Quest Technologies, American). Noise exposure was evaluated with equivalent continuous dB(A)-weighted sound pressure levels (L_Aeq_, 8 h). Besides, the previous recorded data on noise exposure levels of the factory were collected. Cumulative noise exposure (CNE) was calculated to determine the actual noise exposure for each subject based on every period of occupational history, which was defined as [21]:

$$CNE=10\log\left[\frac{1}{T_{ref}}\sum_{i=1}^{n}\left(T_{i}\times 10^{L_{Aeq\cdot 8hi/10}}\right)\right]$$

where L_Aeq,8hi_ is the equivalent continuous A-weighted noise exposure level in decibels normalized to an 8 h working day, occurring over the time interval T_i_ in years, with a total of n different noise level exposure periods (*i.e.*, years spent working in different noise tasks/environments), and T_ref_ = 1 year.

### 2.4. SNP Selection and Genotyping

Informative SNPs were selected based on the HapMap database [22], dBSNP [23] and previous findings from the literature. Since 5’ near gene (as the promoter region), 5′untranslated region (5′UTR), 3′UTR, or coding regions with amino acid variation have the high possibility of functional regions, inclusion criteria were as follows: SNPs located in the entire region of *POU4F3* and *GRHL2* genes with minor allele frequency (MAF) >0.10 in Chinese Han, a linkage disequilibrium (LD) value of r^2^ > 0.80 and/or located in the 5’ near gene, 5’UTR, 3’UTR or coding regions with amino acid changes. In total, 10 single-nucleotide polymorphisms (SNPs) including rs1368402, rs891969 (*POU4F3*), rs611419, rs10955255, rs1981361, rs3779617, rs3735713, rs3824090, rs3735714 and rs3735715 (*GRHL2*) were selected to test the associations with the risk of NIHL.

Genomic DNA was extracted from all blood samples (2 mL) using the standard procedures of the Lifefeng extraction Kit (Shanghai Lifefeng Biotech Co., Ltd., Shanghai, China). The concentration and purity of genomic DNA was detected by using the NanoPhotometer P360 (Shanghai Boyibio Biotech Co., Ltd., Shanghai, China). In addition, genotypes of these 10 loci among 586 samples were determined using the SNP scan^TM^ multiplex SNP genotyping kit (Genesky Biopharm Technology Co., Ltd., Shanghai, China). PCR products were sequenced by ABI3730XL DNA analyzer and results were analyzed by GeneMapper 4.1 software (Applied Biosystems, Foster City, CA, USA). The whole analysis process was performed blind.

### 2.5. Statistical Analysis

Hardy-Weinberg equilibrium (HWE) test was checked for each SNP among control subjects using χ^2^-test. Continuous variables were expressed as mean ± standard deviation (SD), while categorical variables were expressed as frequencies (%). Paired samples *t*-test was used to compare demographic information between case and control group for continuous variables, and the χ^2^-test was used for categorical variables. Adjusted odds ratios (ORs) with 95% confidence intervals (CIs) were computed by conditional logistic regression analysis to test the associations between the genotypes and NIHL risk after adjusting for the potential confounding factors such as smoking, drinking and CNE. Bonferroni correction was performed to control for multiple testing, which resulted in a corrected significance level of *p <* 0.0003. The associations of genotypes with NIHL were stratified by noise exposure levels and CNE. All noise exposed workers were divided into four categories (two noise exposure categories, ≤85 dB(A) and >85 dB(A), as well as two CNE categories ≤95 dB(A) and >95 dB(A)). Haploview version 4.2 software [24] was used to estimate the haplotypes and investigate the LD between the SNPs. All statistical analyses were two-sided by using SPSS 18.0 (IBM SPSS Statistics 18 for Windows, Chicago, IL, USA), and the significance level was set at 0.05.

## 3. Results

### 3.1. Subject Characteristics

The demographic and general characteristics of the subjects were described in Supplementary Table S1. No statistically significant differences were seen between case and control individuals in the distribution of age, tenure, hypertension, drinking status, use of earplugs, noise exposure level and CNE (*p* > 0.05). However, the average of binaural HL of high frequency in the case group was over five times greater than that of the control subjects ((51.4 ± 8.8) dB *vs.* (9.3 ± 9.1) dB, *p* < 0.001). Also, the case group included a significantly higher percentage of smokers (*p* < 0.001) and a higher average height (*p* = 0.026). The variables (smoking, drinking and CNE) were further adjusted in the conditional logistic regression.

### 3.2. Associations of POU4F3 and GRHL2 Variants with the Risk of NIHL

Basic information of the 10 SNPs in the *POU4F3* and *GRHL2* genes and the distributions of the genotypes in the case and control subjects are displayed in Table 1 and Table 2, respectively. All the SNPs in the controls were in Hardy-Weinberg equilibrium (HWE) (*p* > 0.05). The minor allele frequency (MAF) for all SNPs was higher than 10%, indicating that those SNPs were frequent in the Chinese Han population.

After adjusting for smoking, drinking and CNE, subjects carrying the CC genotype of rs1981361 were significantly associated with a higher risk of NIHL (adjusted OR = 1.59; 95% CI: 1.08–2.32, *p =* 0.018) than those carrying CT/TT. For rs3735715, subjects carrying the GG genotype showed more susceptibility to NIHL with an adjusted OR of 1.48 (95% CI = 1.01–2.19, *p* = 0.046) compared with the subjects carrying AA/GA. However, no significant differences were detected in genotypes of the other six SNPs in *GRHL2* or the two SNPs in *POU4F3* between case and control subjects (*p* > 0.05). After applying the Bonferroni correction, the associations were no longer statistically significant.

### 3.3. Interaction and Stratification Analysis of POU4F3 and GRHL2 by Noise Exposure Level and CNE

The results of stratified analysis for interactions between SNPs and noise exposure are shown in Table 3 (presented only for the SNPs with significant results). Four out of the 10 SNPs had significant *p*-values for the higher noise level (noise exposure level > 85 dB(A) or CNE > 95 dB(A)): rs3735715, rs1981361, rs1368402 and rs891969. For rs3735715, when the noise exposure levels > 85 dB(A), compared with the AA/GA genotypes, the GG genotype was significantly more frequent among the case group (*p* = 0.004), which indicated that GG had a causative effect (adjusted OR = 2.27; 95% CI: 1.31–3.96). Similarly, when CNE > 95 dB(A), compared with the AA/GA genotype, subjects with the GG genotype of rs3735715 were more susceptible to NIHL with an adjusted OR of 1.99 (95% CI = 1.20–3.30, *p* = 0.008). For rs1981361, when the noise exposure levels > 85 dB(A), compared with the CT/TT genotypes, the CC genotype increased the risk of NIHL (adjusted OR = 1.92; 95% CI: 1.18–3.11, *p* = 0.008). Moreover, when CNE > 95 dB(A), for rs1368402, comparing with CC/CA genotypes, the AA genotype increased the risk of NIHL (adjusted OR = 1.62; 95% CI = 1.04–2.52, *p* = 0.032); for rs891969, compared with the AA/GA genotypes, the GG genotype was the risk genotype (adjusted OR = 1.65; 95% CI: 1.06–2.57, *p* = 0.026). However, there was no such trend in the lower noise exposure levels or CNE levels. After applying the Bonferroni correction, all the associations were no longer statistically significant.

### 3.4. Association of POU4F3 and GRHL2 Haplotypes with NIHL

Haplotypes were inferred based on observed genotypes by using Haploview software, and a stratification analysis by noise exposure level and CNE was also conducted to explore the interaction between haplotypes and noise exposure. Two SNPs in the *POU4F3* gene (rs1368402 and rs891969) were in linkage disequilibrium (LD) (D′ = 0.982 and r^2^ = 0.994). Pairwise LD between the eight SNPs in the *GRHL2* gene was shown in Supplementary Table S3.

From Table 4, no significant *p*-values were obtained before stratification (*p* > 0.05). However, after stratification, two haplotypes, CA in *POU4F3* and GCCG in *GRHL2*, showed an association with NIHL in the higher noise levels (noise exposure level > 85 dB(A) or CNE > 95 dB(A)). When CNE > 95 dB(A), haplotype CA showed a protective effect on NIHL compared with haplotype AG (adjusted OR = 0.69; 95% CI = 0.48–0.99, *p* < 0.05). Furthermore, when the noise exposure level > 85 dB(A), compared with haplotype GCCA, frequencies of haplotype GCCG were significantly higher in the case group, indicating it to be a risk haplotype (adjusted OR = 1.83; 95% CI = 1.12–2.96, *p* < 0.05). Similarly, when CNE > 95 dB(A), having haplotype GCCA as the reference, subjects with haplotype GCCG were more susceptible to NIHL with an adjusted OR of 1.55 (95% CI = 1.01–2.37, *p* < 0.05). After applying the Bonferroni correction, all the associations were no longer statistically significant.

## 4. Discussion

In this study, 10 SNPs located in the *POU4F3* and *GRHL2* genes, which are regarded as the genes of autosomal dominant deafness, taking into account noise exposure, were analyzed in a Chinese sample set. These genes had previously been reported in noise-exposed workers in Chinese [17,18] and Swedish populations [11] where significant associations with NIHL had been detected. In order to confirm the susceptibility genes for complex diseases, it is important to replicate in independent population. Besides, gene-based replication is consistent with replicating association findings at the SNP or the haplotype level [25]. This study confirmed that the *GRHL2* gene might underlie an increased susceptibility to the development of NIHL. However, the effect of *POU4F3* polymorphisms on NIHL could only be detected when noise exposure was taken into account. Furthermore, several significant interactions were found, including those among four different SNPs and two predictor haplotypes.

The *POU4F3* gene on 5q31 (DFNA15) is one of the two genes belonging to the superfamily of POU domain transcription factors and is expressed specifically in inner-ear hair cells [26]. In this study, we failed to find that the genetic variations of rs1368402 and rs891969 in the *POU4F3* gene were associated with NIHL risk, which corresponded with the finding of the research conducted in the Poland population [11]. These SNPs did not seem to play an important role alone in the development of NIHL. However, considering the race difference in genetic susceptibility and the moderate sample size, more studies are needed to confirm or question these findings.

GRHL2 encodes the Grainyhead-like 2 protein, also known as transcription factor cellular promoter 2-like 3 (TFCP2L3), which is highly expressed in epithelial cells lining the cochlear duct [27,28]. From this study, the significant differences between case and control subjects were seen in the *GRHL2* rs1981361 and rs3735715 variants in the Chinese sample set. Additionally, among noise-exposed subjects, individuals carrying rs1981361 CC genotype and rs3735715 GG genotype were more susceptible to NIHL. A similar result was reported previously that rs1981361 in Sweden was found to be associated with NIHL [11]. On the contrary, Li *et al.* [17] failed to identify the significant association of rs3735715, but found rs611419 AT/TT genotypes conferred protection against NIHL in the Chinese population. This study confirmed the hypothesis that *GRHL2* was an NIHL susceptibility gene, even after adjustment for smoking, drinking and CNE.

NIHL is well known as a multi-factorial disease; thus, related research usually jointly studies the effects of genetic and environmental factors. As noise is the most frequent cause of NIHL, the majority of the studies have already confirmed that candidate genes might affect the susceptibility to NIHL in the higher-noise-exposed population [29,30]. Therefore, this study conducted a stratified association analysis between SNPs and NIHL by the noise exposure levels and CNE. After stratification, interaction effects were detected between genotypes of rs1368402, rs891969, rs3735715 and rs1981361 with noise in the higher level noise exposure groups (noise exposure level > 85 dB(A) or CNE > 95 dB(A)). Similarly, an analysis of the Swedish population showed a positive interaction between rs891969 in the *POU4F3* gene and noise. This is reasonable since the higher the noise level, the more harmful it might be, and consequently the more effect that would be seen [29]. These findings were also in agreement with the previous studies [29,30] and suggested that the interactions between genetic polymorphisms of *POU4F3* and *GRHL2* genes with noise exposure might play important roles in NIHL incidence.

The previous studies have indicated that a differential effect of the haplotype on noise sensitivity might exist according to the noise exposure level [29,31]. This study was the first to perform haplotype analysis on *POU4F3* and *GRHL2* genes to analyze their correlations with NIHL. Odds ratios were calculated in the different noise exposure groups as well. The results showed that protective haplotype CA in *POU4F3* and risk haplotype GCCG in *GRHL2* were significantly different in higher level noise exposure groups (noise exposure level > 85 dB(A) or CNE > 95 dB(A)). Although individual SNP analysis did not show any association between these SNPs and NIHL risk, except for rs3735715, the significant associations were detected after they built the haplotype combined with noise exposure. These indicated that the haplotypes might play a role in the development of NIHL, and higher noise exposure might induce a higher risk. However, these mechanisms require further investigations.

The main strength of this study is that a matched case-control study was performed to provide better efficiency by forcing the case and control subjects to have similar distributions across the basic characteristics and environmental variables. Therefore, the case and control subjects had high comparability in basic and environmental confounders. Secondly, all the experimental (DNA extraction and genotyping) operation personnel and participants were blinded without any knowledge of the disease status in advance, in order to reach a relatively more objective conclusion.

This study also has some limitations that should be considered. Firstly, compared with other studies, the sample size was moderate, which might limit the ability to generalize more findings. However, many of the results shown here add more weight to previously identified genes associated with NIHL. Secondly, when the Bonferroni correction was applied in our study, the significance of the findings was overwhelming. However, it is recognized that replication of findings in independent populations is much more important than obtaining highly significant *p*-values [20]. Finally, the workers might be exposed to noise in other places, such as the community, but this was too complicated to take into consideration.

## 5. Conclusions

Our study indicated that the genetic variation in the *GRHL2* gene might play an important role in determining individual susceptibility to NIHL. However, the effect of *POU4F3* polymorphisms on NIHL could only be detected when taking noise exposure into consideration. Furthermore, the significant interactions between the genetic polymorphisms and environmental factors were detected, which jointly contributed to NIHL. However, when the Bonferroni correction applied, these differences were no longer significant. The results should be confirmed in large population-based prospective studies in the future. Further genetic and functional analysis is warranted to explain the potential mechanism of genetic variation in the development of NIHL.

## Figures and Tables

**Table 1:** Basic information of the 10 SNPs in *POU4F3* and *GRHL2* genes.

Genes	SNPs	Minor/Major Allele(A1/A2)	Location	MAF			*p (HWE) ^a^*	A1A1/A1A2/A2A2	
				HapMap	Case	Control		Case	Control
*POU4F3*	rs1368402	C/A	5′_flanking	0.277	0.207	0.230	0.751	12/75/150	14/82/141
	rs891969	A/G	3′_flanking	0.275	0.205	0.228	0.687	12/74/153	14/81/142
*GRHL2*	rs611419	A/T	5′_flanking	0.356	0.446	0.423	0.367	56/101/79	47/108/81
	rs10955255	G/A	Intron1	0.161	0.241	0.247	1.000	14/87/139	14/90/135
	rs1981361	T/C	Intron1	0.244	0.249	0.295	0.171	20/79/136	16/109/112
	rs3779617	A/G	Exon9	0.127	0.087	0.105	0.058	0/42/195	6/38/195
	rs3735713	A/G	3'UTR	0.301	0.280	0.276	0.209	19/96/121	14/104/118
	rs3824090	T/C	3'UTR	0.234	0.190	0.201	0.081	6/79/151	5/86/145
	rs3735714	T/C	3'UTR	0.389	0.349	0.360	0.073	28/111/100	24/124/91
	rs3735715	A/G	3'UTR	0.427	0.435	0.477	0.138	52/104/80	49/130/56

Note: SNP: single-nucleotide polymorphisms; MAF: minor allele frequency; HWE: Hardy-Weinberg equilibrium **^a^** HWE tests were performed using the χ^2^ test for each SNP among control subjects.

**Table 2:** Associations of candidate SNPs with the risk of NIHL.

Genes	SNPs	Genotypes	Case (*n* = 239)		Control (*n* = 239)		Adjusted *OR* (95% *CI*) *	p *
			N	%	N	%		
*POU4F3*	rs1368402	CC/CA	87	36.4	96	40.2	1.00	
		AA	150	62.8	141	59.0	1.26 (0.87–1.84)	0.223
	rs891969	AA/GA	86	36.0	95	39.7	1.00	
		GG	153	64.0	142	59.4	1.23 (0.84–1.79)	0.283
*GRHL2*	rs611419	AA/AT	157	65.7	155	64.9	1.00	
		TT	79	33.1	81	33.9	0.92 (0.60–1.39)	0.676
	rs10955255	GG/AG	101	42.3	104	43.5	1.00	
		AA	139	58.2	135	56.5	1.13 (0.76–1.66)	0.551
	rs1981361	CT/TT	99	41.4	125	52.3	1.00	
		CC	136	56.9	112	46.9	**1.59 (1.08–2.32)**	**0.018**
	rs3779617	AA/GA	42	17.6	44	18.4	1.00	
		GG	195	81.6	195	81.6	1.09 (0.69–1.73)	0.715
	rs3735713	AA/GA	115	48.1	118	49.4	1.00	
		GG	121	50.6	118	49.4	0.99 (0.68–1.44)	0.973
	rs3824090	TT/CT	85	35.6	91	38.1	1.00	
		CC	151	63.2	145	60.7	1.13 (0.75–1.69)	0.560
	rs3735714	TT/CT	139	58.2	148	61.9	1.00	
		CC	100	41.8	91	38.1	1.17 (0.79–1.72)	0.436
	rs3735715	AA/GA	156	65.3	179	74.9	1.00	
		GG	80	33.5	56	23.4	**1.48 (1.01–2.19)**	**0.046**

Notes * Adjusted for smoking, drinking and CNE.

**Table 3:** Stratified analysis of associated SNPs by noise exposure level or CNE.

Variables	SNPs	Genotypes	Case (n, %)	Control (n, %)	Adjusted OR (95% CI) *	p *
**Noise Exposure Level (dB(A))**						
≤85	rs3735715	AA/GA	66 (71.0)	68 (69.4)	1.00	
		GG	27 (29.0)	30 (30.6)	0.92 (0.49–1.76)	0.808
>85		AA/GA	90 (62.9)	111 (81.0)	1.00	
		GG	53 (37.1)	26 (19.0)	**2.27 (1.31–3.96)**	**0.004**
≤85	rs1981361	CT/TT	41 (44.6)	47 (48.5)	1.00	
		CC	51 (55.4)	50 (51.5)	1.18 (0.65–2.12)	0.587
>85		CT/TT	58 (40.6)	78 (55.7)	1.00	
		CC	85 (59.4)	62 (44.3)	**1.92 (1.18–3.11)**	**0.008**
**CNE (dB(A))**						
≤95	rs3735715	AA/GA	43 (68.3)	40 (63.5)	1.00	
		GG	20 (31.7)	23 (36.5)	0.80 (0.38–1.70)	0.561
>95		AA/GA	113 (65.3)	139 (80.8)	1.00	
		GG	60 (34.7)	33 (19.2)	**1.99 (1.20–3.30)**	**0.008**
≤95	rs1368402	CC/CA	24 (38.1)	16 (25.0)	1.00	
		AA	39 (61.9)	48 (75.0)	0.53 (0.25–1.16)	0.113
>95		CC/CA	63 (36.2)	80 (46.2)	1.00	
		AA	111 (63.8)	93 (53.8)	**1.62 (1.04–2.52)**	**0.032**
≤95	rs891969	AA/GA	25 (39.7)	16 (25.4)	1.00	
		GG	38 (60.3)	47 (74.6)	0.50 (0.23–1.08)	0.078
>95		AA/GA	61 (34.7)	79 (45.4)	1.00	
		GG	115 (65.3)	95 (54.6)	**1.65(1.06–2.57)**	**0.026**

Notes * Adjusted for smoking and drinking.

**Table 4:** Assessment of association between the haplotypes and NIHL.

Genes	Haplotypes	Case (n, %)	Control (n, %)	Total	Noise Exposure Level ≤ 85 dB(A)	Noise Exposure Level > 85dB(A)	CNE ≤ 95 dB(A)	CNE > 95 dB(A)
				Adjusted *OR* (95%) *				
*POU4F3*	AG	378 (79.1)	368 (77.0)	1.00	1.00	1.00	1.00	1.00
	CA	97 (20.3)	110 (23.0)	0.90 (0.70–1.16)	0.88 (0.51–1.51)	0.86 (0.58–1.27)	1.72 (0.87–3.38)	**0.69 (0.48–0.99)**
*GRHL2*	GCCA	212 (44.4)	232 (48.5)	1.00	1.00	1.00	1.00	1.00
	GCCG	99 (20.7)	74 (15.5)	1.20 (0.92–1.59)	0.96 (0.55–1.67)	**1.83 (1.12–2.96)**	0.10 (0.50–2.01)	**1.55 (1.01–2.37)**
	ATTG	92 (19.2)	97 (20.3)	1.02 (0.77–1.35)	0.86 (0.49–1.49)	1.11 (0.71–1.73)	0.91 (0.46–1.78)	1.06 (0.70–1.59)
	ATCG	43 (9.0)	37 (7.7)	1.20 (0.82–1.77)	1.09 (0.52–2.29)	1.46 (0.77–2.79)	0.76 (0.34–1.71)	1.69 (0.91–3.15)

Notes: Haplotype analysis was restricted to the SNPs that were in strong LD (D’ > 0.98); haplotypes of *POU4F3* were deduced for the following SNPs: rs1368402 and rs891969; haplotypes of *GRHL2* were deduced for the following SNPs: rs3735713, rs3824090, rs3735714 and rs3735715; * adjusted for smoking and drinking; only haplotype with frequency >5% was shown in this table; bold signifies *p* < 0.05.

## Supplementary Materials

The following are available online at www.mdpi.com/1660-4601/13/6/561/s1, Table S1: Demographic and general characteristics of case and control subjects, Table S2: Associations of candidate SNPs with the risk of NIHL, Table S3: Linkage disequilibrium test of GRHL2 gene.

## Abbreviations

NIHL	noise-induced hearing loss
POU4F3	POU Domain, Class 4, Transcription Factor 3
GRHL2	grainyhead-like2
SNPs	single nucleotide polymorphisms
HWE	hardy-Weinberg equilibrium
MAF	minor allele frequency
LD	linkage disequilibrium
CHB	han Chinese individuals from Beijing
PTA	pure-tone audiometry
HL	hearing level
OR	odds ratio
CI	confidence interval
