# Supplementary material for: Genetic Variation in POU4F3 and GRHL2 Associated with Noise-Induced Hearing Loss in Chinese Population: A Case-Control Study
Source: Int J Environ Res Public Health. 2016 Jun 3;13(6):561. doi: 10.3390/ijerph13060561 (PMC4924018; doi:10.3390/ijerph13060561)
Supplement: Supplementary file 1 [file ijerph-13-00561-s001.pdf]

Genetic Variation in *POU4F3* and *GRHL2* Associated with Noise-Induced Hearing Loss in Chinese Population: A Case-Control Study

Xiangrong Xu, Qiuyue Yang, Jie Jiao, Lihua He, Shanfa Yu, Jingjing Wang, Guizhen Gu, Guoshun Chen, Wenhui Zhou, Hui Wu, Yanhong Li and Huanling Zhang

Table S1. Demographic and general characteristics of case and control subjects.

Variables	Case (n = 239)	Control (n = 239)	p
Age (yr), mean ± SD	40.4 ± 8.3	39.5 ± 8.2	0.264
Tenure (yr), mean ± SD	18.7 ± 9.2	18.1 ± 8.9	0.447
HL (dB), mean ± SD	51.4 ± 8.8	9.3 ± 9.1	**<0.001**
Height (cm),mean ± SD	171.2 ± 5.5	170.1 ± 5.4	**0.026**
Hypertension, n (%)			
yes	107 (44.8)	100 (41.8)	0.518
no	132 (55.2)	139 (58.2)	
Smoking, n (%)			
yes	161 (67.4)	118 (49.4)	**<0.001**
no	78 (32.6)	121 (50.6)	
Drinking (Alcohol), n (%)			
yes	170 (71.1)	166 (69.5)	0.689
no	69 (28.9)	73 (30.5)	
Protector (Eaeplug), n (%)			
yes	125 (52.3)	118 (49.4)	0.522
no	114 (47.7)	121 (50.6)	
Noise intensity exposure, dB(A)	86.1 ± 3.9	86.1 ± 3.9	0.930
CNE, dB(A)	98.2 ± 4.6	98.1 ± 4.5	0.856

Notes: HL: hearing level; CNE: cumulative noise exposure.

Table S2. Associations of candidate SNPs with the risk of NIHL.

Genes	SNPs	Genotypes	Case (n = 239)		Control (n = 239)		Adjusted *OR* (95% *CI*) *	p *
			N	%	N	%		
POU4F3	rs1368402	CC	12	5.0	14	5.9	1.00	
		CA	75	31.4	82	34.3	0.87 (0.37–2.07)	0.756
		AA	150	62.8	141	59.0	1.13 (0.49–2.57)	0.780
		CA/AA	225	94.1	223	93.3	1.13 (0.51–2.51)	0.762
		CC/CA/AA						0.304
	rs891969	AA	12	5.0	14	5.9	1.00	
		GA	74	31.0	81	33.9	0.91 (0.39–2.15)	0.837
		GG	153	64.0	142	59.4	1.14 (0.50–2.60)	0.759
		GA/GG	227	95.0	223	93.3	1.06 (0.47–2.38)	0.895
		AA/GA/GG						0.354
GRHL2	rs611419	AA	56	23.4	47	19.7	1.00	
		AT	101	42.3	108	45.2	0.77 (0.47–1.26)	0.301

Table S2. *Cont.*

Genes	SNPs	Genotypes	Case (*n* = 239)		Control (*n* = 239)		Adjusted *OR* (95% *CI*) *	*p* *
			N	%	N	%		
		TT	79	33.1	81	33.9	0.77 (0.46–1.31)	0.336
		AT/TT	180	75.3	189	79.1	0.77 (0.49–1.22)	0.265
		AA/AT/TT						0.365
	rs10955255	GG	14	5.9	14	5.9	1.00	
		AG	87	36.4	90	37.7	1.10 (0.48–2.53)	0.822
		AA	139	58.2	135	56.5	1.22 (0.55–2.71)	0.627
		AG/AA	226	94.6	225	94.1	1.18 (0.54–2.58)	0.684
		GG/AG/AA						0.524
	rs1981361	TT	20	8.4	16	6.7	1.00	
		CT	79	33.1	109	45.6	0.63 (0.30–1.34)	0.231
		CC	136	56.9	112	46.9	1.09 (0.53–2.24)	0.811
		CT/CC	215	90.0	221	92.5	0.90 (0.45–1.82)	0.774
		TT/CT/CC					1.30 (0.97–1.74)	0.085
	rs3779617	AA	0	0.0	6	2.5	1.00	
		GA	42	17.6	38	15.9	–	0.812
		GG	195	81.6	195	81.6	–	0.812
		GA/GG	237	99.2	233	97.5	–	0.976
		AA/GA/GG						0.324
	rs3735713	AA	19	7.9	14	5.9	1.00	
		GA	96	40.2	104	43.5	0.62 (0.27–1.44)	0.268
		GG	121	50.6	118	49.4	0.64 (0.27–1.52)	0.312
		GA/GG	217	90.8	222	92.9	0.64 (0.28–1.44)	0.273
		AA/GA/GG						0.645
	rs3824090	TT	6	2.5	5	2.1	1.00	
		CT	79	33.1	86	36.0	0.67 (0.17–2.66)	0.568
		CC	151	63.2	145	60.7	0.77 (0.20–3.03)	0.707
		CT/CC	230	96.2	231	96.7	0.73 (0.19–2.81)	0.642
		TT/CT/CC						0.684
	rs3735714	TT	28	11.7	24	10.0	1.00	
		CT	111	46.4	124	51.9	0.75 (0.39–1.44)	0.389
		CC	100	41.8	91	38.1	0.92 (0.47–1.80)	0.799
		CT/CC	211	88.3	215	90.0	0.81 (0.43–1.53)	0.521
		TT/TC/CC						0.769
	rs3735715	AA	52	21.8	49	20.5	1.00	
		GA	104	43.5	130	54.4	0.78 (0.49–1.25)	0.304
		GG	80	33.5	56	23.4	1.23 (0.73–2.09)	0.443
		GA/GG	184	77.0	186	77.8	0.91 (0.58–1.48)	0.678
		GG/GA/AA						0.265

Note: * Adjusted for smoking, drinking and CNE.

Table S3. Linkage disequilibrium test of GRHL2 gene.

SNPs	rs611419	rs10955255	rs1981361	rs3779617	rs3735713	rs3824090	rs3735714	rs3735715
**rs611419**	–	0.033	0.139	0.177	0.016	0.095	0.023	0.015
**rs10955255**	0.000	–	0.792	0.009	0.332	0.262	0.375	0.198
**rs1981361**	0.006	0.525	–	0.039	0.214	0.098	0.193	0.070
**rs3779617**	0.003	0.000	0.000	–	0.398	0.58	0.493	0.420
**rs3735713**	0.000	0.014	0.007	0.007	–	1.000	1.000	0.989
**rs3824090**	0.002	0.005	0.001	0.009	0.630	–	1.000	0.981
**rs3735714**	0.000	0.025	0.008	0.014	0.713	0.449	–	0.992
**rs3735715**	0.000	0.014	0.002	0.022	0.331	0.205	0.466	–

Notes: The upper triangle was D′ value and the lower triangle was r^2 value.

© 2016 by the authors; licensee MDPI, Basel, Switzerland. This article is an open access article distributed under the terms and conditions of the Creative Commons by Attribution (CC-BY) license (http://creativecommons.org/licenses/by/4.0/).
